# Editorial: Immersive technology and ambient intelligence for assistive living, medical, and healthcare solutions

**DOI:** 10.3389/fnhum.2024.1376959

**Published:** 2024-02-21

**Authors:** Omneya Attallah, Ahmad Al-Kabbany, Shaza B. Zaghlool, Mohamed Kholief

**Affiliations:** ^1^Department of Electronics and Communications Engineering, College of Engineering and Technology, Arab Academy for Science, Technology, and Maritime Transport, Alexandria, Egypt; ^2^Intelligent Systems Lab, Arab Academy for Science, Technology, and Maritime Transport, Alexandria, Egypt; ^3^Department of Research and Development, VRapeutic Inc., Ottawa, ON, Canada; ^4^Department of Biophysics and Physiology, Weill Cornell Medicine - Qatar, Doha, Qatar; ^5^Computer Science and Information Systems, College of Computing and Information Technology, Arab Academy for Science, Technology and Maritime Transport, Alexandria, Egypt

**Keywords:** artificial intelligence, immersive technology, ambient intelligence, brain computer inteface, biomedical signal processing

## 1 Introduction

The growing elderly population is a significant socioeconomic concern in the modern era. By the year 2030, the WHO predicts that one out of every six individuals will be over the age of 60 (Lee et al., 2023). The increasing number of elderly people presents increasingly complex and unpredictable needs that are difficult to meet with conventional healthcare facilities. The majority of traditional healthcare services rely on in-person interactions and human involvement. In the meantime, the majority of countries depend heavily on healthcare providers and nursing professionals. Nevertheless, as the percentage of older people in the community rises and the need for wellbeing among the elderly grows, novel ideas for effective and efficient delivery of healthcare, based on everyday care for the elderly persons, assisted with modern digital technologies have become essential (Evangelista et al., 2019). Nevertheless, the discrepancy between the scarcity and inequitable allocation of medical healthcare on one side, and the escalating demands for health-related services on another side, is transforming into an ever more evident conflict (Chen et al., 2010).

WHO reports that in addition to an aging population, the incidence of chronic illnesses is rising quickly globally and that new risks are emerging, including rising expenses, a shortage of medical professionals, and the accessibility to scarce resources. Collectively, such obstacles will exert a significant burden on the effectiveness and cost-effectiveness of healthcare systems worldwide. Furthermore, these prevailing concerns have prompted the necessity of bringing healthcare services directly to homes (Ahmad et al., 2022; Alshamrani, 2022). Additionally, the worldwide healthcare industry and its associated employees, infrastructures, as well as supply chain management, have been subjected to tremendous stress as a result of the latest COVID-19 outbreak. The COVID-19 pandemic has been the main catalyst for driving swift transformation throughout the healthcare industry, prompting stakeholders to actively embrace technological advancements in healthcare. Studies have revealed that emerging technologies have had significant potential and central role in the aftermath of the pandemic, leading to their adoption as a new standard way of life (Alghamdi and Alghamdi, 2022; Chandra et al., 2022). Hence, cutting-edge technologies such as Artificial Intelligence (AI), wearable devices, ambient assistive technology, immersive technologies, and the Internet of Things (IoT) have recently had a growing importance in the healthcare sector.

Significant and fundamental transformations have occurred in the healthcare sector since the conclusion of the global epidemic of COVID-19. Implementing innovative healthcare services based on new digital technologies has been proven efficient in guaranteeing optimal treatment results, cost-effectiveness, and long-term viability of healthcare systems. Emerging technologies are facilitating the provision of safe and secure healthcare for patients in distant areas (Lee and Lee, 2021; Chengoden et al., 2023). An illustration of such technologies is the IoT, which has become remarkably important in the field of healthcare, especially in remote settings (Qadri et al., 2020). This has resulted in the emergence of the idea of the Internet of Medical Things (IoMT). Moreover, the remote monitoring of patients and the quality of life of individuals can be enhanced through the use of wearable technologies for ambient assisted living, such as smartwatches and bracelets, wearable electroencephalograms (EEG), portable electrocardiograms (ECG), and wearable electromyograms (EMG), etc. Moreover, the utilization of immersive technologies, including virtual, augmented, and mixed reality technologies using smart goggles, can elicit a sense of being in a care unit and thus have a profound effect on healthcare, encompassing both mental and physical aspects. They can assist medical experts in efficiently strategizing and diagnosing illnesses as well as facilitating rehabilitation procedures. Furthermore, the integration of Artificial Intelligence (AI) into cutting-edge devices and equipment has facilitated the automation of crucial services and augmented human intelligence through the utilization of extensive data (Wang et al., 2018).

The primary objective of this Research Topic is to showcase research papers in the cross-disciplinary areas of immersive technologies, ambient assistive technology, and artificial intelligence as they relate to medical, healthcare, and assistive living applications. Additionally, it seeks to increase and emphasize the utilization of state-of-the-art technologies in solving problems related to medicine, healthcare services, and public health, and show how these technologies have improved healthcare systems.

## 2 Summary of the papers submitted to the Research Topic

Every paper submitted to the Research Topic passed a meticulous review procedure, which included a minimum of two reviewers and two rounds of comprehensive revisions prior to acceptance. Four papers were chosen, each representing original research. The articles that have significantly advanced this Research Topic are enumerated below.

In this Research Topic, Maidhof et al. stated that video-based ambient assisted living (AAL) technologies offer a novel method for helping elderly individuals maintain their independence and autonomy within their residences. Such visual aids possess the capacity to enhance security, perceived safety, and reassurance for families and carers by recognizing various emergencies or critical health conditions. While video-based technologies offer various possibilities and benefits, their use to observe everyday activities raises concerns regarding privacy infringement and data protection. In order to achieve a sustainable design and successful implementation of these technological advancements, it is necessary to conduct a comprehensive assessment of the future users' willingness to adopt them. This assessment should consider factors such as the perceived advantages and obstacles. Additionally, it is crucial to consider the potential impact on privacy and the specific requirements related to filming various events. Hence, their research examined the level of acceptance and perception of the benefits and barriers associated with the utilization of video-based AAL technologies for various daily activities. This was done through an online survey that employed scenarios, with a total of 146 participants.

The initial analysis of Maidhof et al. study, revealed clear assessment criteria for 25 daily activities, categorizing them into two groups based on their privacy requirements: activities that have significant privacy requirements (such as changing clothes and showering) as well as tasks that have minor privacy demands (such as gardening, eating, and drinking). Next, a comparison was made between three sample kinds of activities in terms of their acceptance, perceived benefits, and barriers. The adoption and perceived advantages of utilizing video-based AAL technologies were found to be greater in home and social contexts as opposed to intimate activities. The most robust obstacle to perception was observed concerning intimate activities, primarily concerning privacy concerns.

This Research Topic also involves the work of Zhou et al. who were concerned with evaluating the processing speed of individuals (which refers to the capacity to rapidly process information). It is commonly recognized as one of the cognitive functions impacted by both multiple sclerosis and schizophrenia. Processing speed is typically evaluated using conventional paper-and-pencil tests. Nevertheless, the administration of these tests typically requires the supervision of medical professionals in a controlled setting, thereby restricting their use for evaluating cognitive abilities or enhancing daily life activities. Thus, Zhou et al. presented a novel approach for assessing processing speed, aimed at aiding clinicians in their diagnostic process. The authors developed a virtual reality street environment that includes the Stroop task, providing a highly engaging experience (referred to as VR-Street). The behavior and performance data were acquired through the execution of a dual-task involving street-crossing and the Stroop test. A dataset consisting of fifty participants was created, with each participant's performance being evaluated using a standardized scale. The Pearson correlation coefficient was employed to determine the association among the dual-task attributes and the cognitive assessment outcomes. Subsequently, a machine learning-based intelligent assessment model was constructed. The findings of this study demonstrated that the dual-task approach employed in this research effectively engaged participants' attention as well as cognitive abilities, providing a more comprehensive representation of their cognitive processing pace. The suggested approach offered a novel avenue for precise quantitative assessment of cognitive ability using virtual reality.

On the other hand, the Research Topic includes the study conducted by McCracken et al.. The study declared that individuals diagnosed with Attention-Deficit/Hyperactivity Disorder (ADHD) frequently experience difficulties in motor functioning and coordination when engaging in activities that involve the adjustment of force. This research offered valuable insights into the impact of modified neural processing and sensorimotor integration (SMI) on a motor learning framework that demands proprioception and force modulation. Prior studies have hypothesized that individuals with ADHD may exhibit modified SMI. These findings can contribute to our comprehension of the neurological function underlying SMI. Thus, in this work, a group of 15 adults diagnosed with ADHD and a control group of 15 neurotypical individuals participated in a unique task called force-matching. In this task, individuals employed their right thumb to match a trace pattern that ranged from 2% to 12% of their greatest voluntary contraction of the Abductor Pollicis Brevis muscle. Furthermore, the participants were also required to undertake a retention test after a period of 24 h. Pre and post-motor acquisition, somatosensory-evoked potentials (SEPs) of the median nerve were collected. EEG (electroencephalogram) was recorded from 1000 trials using a 64-electrode EEG system. The changes in SEP amplitude were adjusted to the baseline values of each participant for that specific peak.

Distinct neural variations were observed among different groups following the collection of a new force-matching motor framework, specifically concerning the N18 peak. The N18 variations indicate that individuals with ADHD experience a decrease in olivary-cerebellar-M1 inhibition while acquiring a new motor skill that relies on force modulation. This may be attributed to challenges in combining the necessary sensory input to successfully execute the task. The findings of this study offer proof that adolescents with ADHD exhibit modified proprioceptive processing while acquiring a new motor skill, in comparison to individuals without ADHD.

Finally, the paper submitted to the Research Topic by Feng et al. revealed that the limited amount of training samples of motor imagery electroencephalogram (MI-EEG) for each person, in addition to the significant variations in MI-EEG across distinct people, can result in poor generalization and low performance of the model for a particular MI activity. Therefore, to address these challenges, a novel approach was suggested. The authors employed an adaptable cross-subject transfer learning technique built upon the transfer learning adaptive boosting (TrAdaBoost) and kernel mean matching (KMM) method. To validate the efficacy and practicability of the suggested approach, the approach was implemented on both BCI Competition IV datasets and in-house datasets. In contrast to the current state of the art, the proposed approach significantly outperformed the performance of MI-EEG signal analysis. Simultaneously, this work also when tested on the in-house dataset, confirmed once again the efficacy of the method. The findings of this research hold clinical relevance for brain rehabilitation.

## 3 Conclusion

The papers in this Research Topic demonstrate substantial advancements in the establishment of cross-disciplinary studies. Specifically, they promote collaborative endeavors originating from the domains of ambient assistive technology, immersive technology, artificial intelligence, machine learning, deep learning, signal processing, healthcare solutions, and medicine. The articles also emphasize the possibility of further advancement in these fields, as well as at the intersection of any relevant fields. We anticipate tremendous strides and accumulations of scientific findings, software platforms, and data if forthcoming research concentrates on how to best serve the healthcare industry and, above all else, individuals via novel artificial intelligence-driven applications. When they are brought together, their collective influence will have a substantial effect on society and hold great scientific significance.

## Author contributions

OA: Writing—original draft, Writing—review & editing. AA-K: Writing—review & editing. SZ: Writing—review & editing. MK: Writing—review & editing.
